# A preliminary metabolites identification of a novel compound with β-adrenolytic activity

**DOI:** 10.1007/s43440-021-00273-9

**Published:** 2021-05-29

**Authors:** Maria Walczak, Joanna Suraj-Prażmowska, Kamil Kuś, Agnieszka Kij, Grażyna Groszek

**Affiliations:** 1 Chair and Department of Toxicology, Jagiellonian University Medical College, Medyczna 9, 30-688 Krakow, Poland; 2 Jagiellonian Centre for Experimental Therapeutics, Jagiellonian University, Bobrzynskiego 14, 30-348 Krakow, Poland; 3 Department of Industrial and Materials Chemistry, Faculty of Chemistry, Rzeszow University of Technology, 6 Powstancow Warszawy Ave, 35-959 Rzeszow, Poland

**Keywords:** Aminopropan-2-ol compound, Metabolites, Renal clearance, Mass spectrometry, Orbitrap, Electrochemistry

## Abstract

**Background:**

The identification of main metabolites and assessment of renal excretion of a novel compound with β-adrenolytic activity (2*RS*)-1-(1*H*-indol-4-yloxy)-3-((2-(2-methoxyphenoxy)ethyl)amino)propan-2-ol, briefly called (*RS*)-9 or 2F109, were studied in vivo in rat serum, urine, faeces, liver, intestine, lungs and kidneys, and in vitro in rat liver microsomes.

**Methods:**

Structures of the metabolites have been developed by comparing the high-resolution product ion mass spectra of metabolites and the parent compound based on the differences in mass values of main fragments. Quantitative analysis of (*RS*)-9 was done using a system of liquid chromatography coupled with a triple quadrupole mass spectrometer API 2000. Identification studies of predicted metabolites were made by a high-resolution mass spectrometer LTQ XL Orbitrap Discovery and using a Roxy^™^ system, for online electrochemical mimicry of oxidative metabolism by cytochrome P450s connected to QTRAP 5500.

**Results:**

For (*RS*)-9 (*m/z* 357.2084) phase I metabolites derived from oxidation process: hydroxyl derivatives (*m/z* 373.2470) and dihydroxyl derivatives (*m/z* 389.4318), and phase II metabolites: *N*-methylated compound (*m/z* 371.1612), *O*-glucuronide (*m/z* 533.5118), and sulfate (*m/z* 437.2350) were identified.

**Conclusion:**

(*RS*)-9 was extensively metabolised to several phase I and II metabolites, and renal excretion was a minor route in its elimination.

**Graphic abstract:**

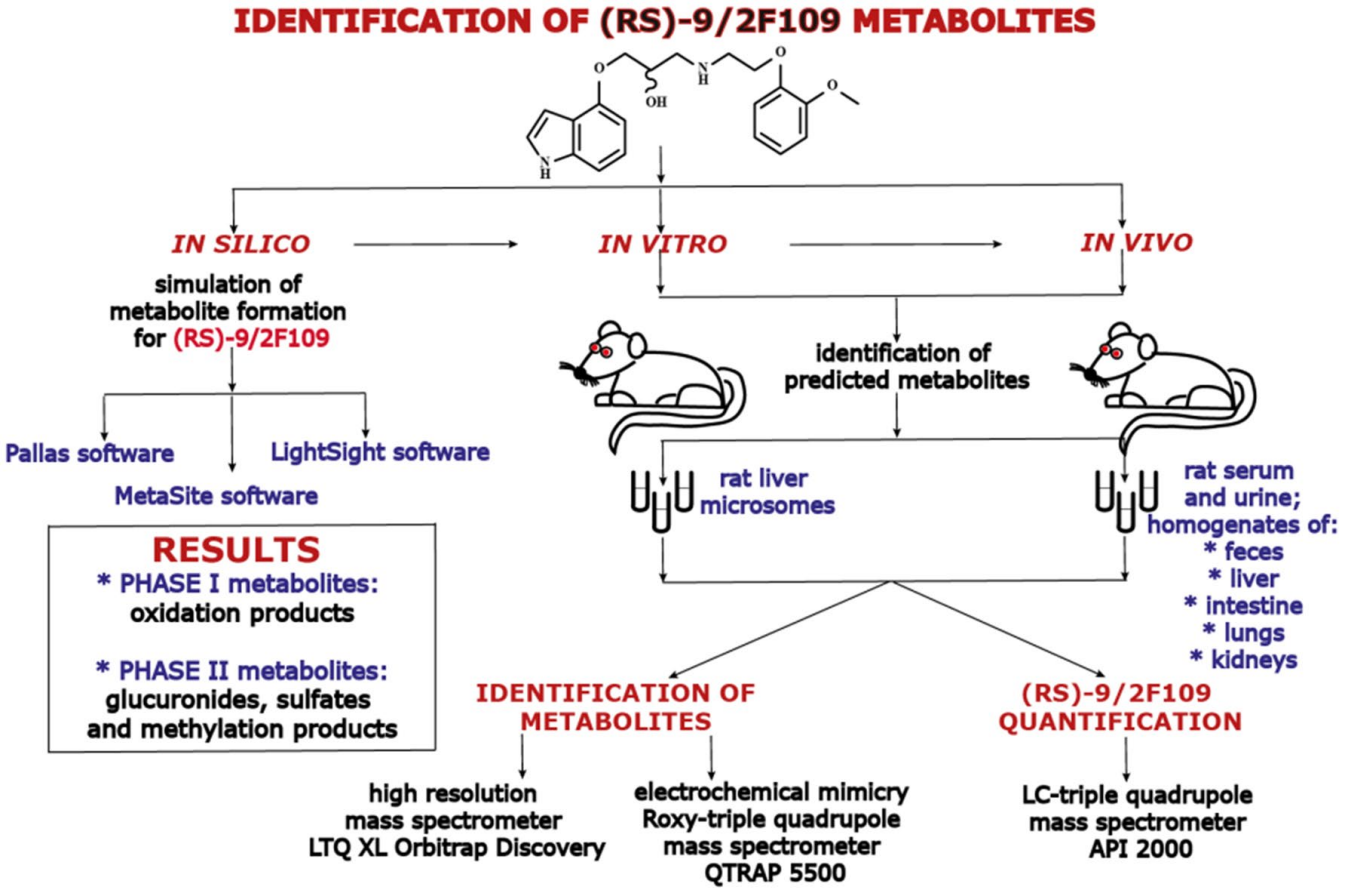

## Introduction

Among the ADME (absorption, distribution, metabolism, and excretion) properties, metabolites’ identification constitutes an important paradigm in the early phase of drug discovery and development [1]. Biotransformation of xenobiotics is an important factor affecting the therapeutic and toxic profile that leads not only to detoxification but sometimes also to the bioactivation of the molecule [2].

The metabolism of xenobiotics is divided into two phases carried out by unique sets of metabolic enzymes. Phase I metabolism involves primarily the functionalisation processes (oxidation, reduction, hydrolysis, chiral inversion, and demethylation) and phase II metabolism is often related to conjugation reactions (glucuronidation, sulfation, methylation, acetylation, condensation, and glutathione conjugation) [3].

To characterise the metabolic profile of new chemical entities (NCEs), it is necessary to identify the major metabolites. Due to the ever-increasing quantity of NCEs’ fast, reliable and high-throughput screening techniques, *e.g*., high-resolution mass spectrometry (HRMS) and electrochemical mimicry play an essential role in the process of evaluation of new drug candidates allowing the elimination of many compounds at the early stages of the drug discovery and limiting the costs of the new drug development [4].

Along with the progress of civilisation, there is an increasing incidence of cardiovascular diseases which along with cancer, diabetes and the present coronavirus disease 2019 (COVID-19) are the leading cause of mortality in the world [5].

According to the World Health Organization (WHO), about 600 million people suffer from hypertension, of which only 20% undergo treatment. Appropriate control of blood pressure is achieved in only 12% of treated patients. For this reason, many research centres are looking for new compounds or derivatives of existing drugs that would enhance the effectiveness and safety of treatment and improve the quality of patients’ lives [6].

Currently, no effective drugs are available for COVID-19 patients, but many drugs have been proposed, such as β-adrenergic blockers which work by decreasing the SARS-CoV-2 entry into the cells by downregulating angiotensin-converting enzyme 2 (ACE2) receptor [7]. In this respect, it is proposed that β-adrenergic blockers may be beneficial in COVID-19 patients with hypertension comorbidity, and in low doses in COVID-19 patients with normal blood pressure [8, 9].

Of particular note are β-blockers of the third generation, multifunctional hybrid drugs, such as nebivolol, carvedilol, celiprolol, and labetalol. Each of them undergoes extensive first-pass metabolism and produces active β-blocking hydroxylated metabolites [10, 11].

In search of NCEs among aminopropan-2-ol derivatives, a new compound (2*RS*)-1-(1*H*-indol-4-yloxy)-3-((2-(2-methoxyphenoxy)ethyl)amino)propan-2-ol in short called (*RS*)-9 or in other studies 2F109 was synthesised by Groszek from the Rzeszow University of Technology [12]. (*RS*)-9 as an analogue of carvedilol and pindolol with fragment structure of these drugs, linked by an aminopropanol moiety, was discovered as a potential drug for the cardiovascular system and is covered by patent claim [13].

An important reason to take into account the identification of (*RS*)-9 metabolites concerns the results of pharmacodynamic and pharmacokinetic studies which showed that (*RS*)-9 has high α_1_- and β_1_-adrenolytic activity with antiarrhythmic and hypotensive effect weaker compared to carvedilol but more active in comparison to propranolol [12]. The pharmacological effect of the compound was evident despite the relatively moderate oral bioavailability (*ca* 40%) [14], moderate binding to blood proteins (*ca* 45%) [15], and stereoselective pharmacokinetics [16]. In terms of the stated purpose of the paper, we believe that the identification of (*RS*)-9 metabolites will be helpful in better understanding and explaining its bioavailability as a result of incomplete absorption or presumably an extensive presystemic metabolism in the first-pass effect.

## Materials and methods

### Chemicals and reagents

(2*RS*)-1-(1*H*-indol-4-yloxy)-3-((2-(2-methoxyphenoxy)ethyl)amino)propan-2-ol ((*RS*)-9, purity > 99.8%) was synthesised in the Department of Industrial and Materials Chemistry, Faculty of Chemistry, Rzeszow University of Technology. Acebutolol (AC) and dextrorphan used as the internal standards, dimethyl sulfoxide, nicotinamide adenine dinucleotide phosphate (NADPH), magnesium chloride, Folin and Ciocalteu’s phenol reagent, potassium sodium tartrate tetrahydrate, cooper sulfate, sucrose, tris(hydroxymethyl)aminomethane (TRIS), sodium phosphate dibasic and potassium chloride were purchased from Sigma-Aldrich (St. Louis, MO, USA). Chemicals such as HPLC grade acetonitrile, methanol, and ethyl acetate were supplied by Merck (Darmstadt, Germany). Formic acid was obtained from Fluka (Buchs, Switzerland). Potassium dihydrogen phosphate, sodium chloride, and acetone were purchased from J.T. Baker (Phillipsburg, PA, USA). Purified water (18.2 MΩ) was delivered by a Milli-Q water system (Millipore, Billerica, MA, USA).

### Instrumentation

Quantitative analyses were performed on an Applied Biosystems/MDS Sciex (Concord, Ontario, Canada) API 2000 triple quadrupole mass spectrometer equipped with an electrospray ionisation interface. The instrument was coupled to an Agilent 1100 (Agilent Technologies, Waldbronn, Germany) HPLC system. Data acquisition and processing were accomplished using Sciex Analyst 1.4.2 data collection and integration software.

A high-resolution LTQ XL Orbitrap Discovery mass spectrometer (Thermo Scientific, Bremen, Germany) equipped with an electrospray ionisation probe was used for metabolite identification in the FT/MS and fragmentation mode at a resolution of 30,000. Data were processed using Xcalibur software.

The electrochemical simulation of metabolites formation by redox reactions was done using the electrochemical system Roxy^™^ (Antec, Leyden, The Netherlands) controlled by Dialog software. For the generation of mass voltammograms and ion spectra of oxidation products, the electrochemical cell was directly coupled with a QTRAP 5500 mass spectrometer (Sciex, Framingham, MA, USA).

### Preparation of stock and working solutions

The stock solution (1.0 mg/mL ± 0.1) was prepared by dissolving an accurately weighed quantity of (*RS*)-9 in methanol. A working solution of (*RS*)-9 at concentrations ranging from 20 ng/mL to 20,000 ng/mL was prepared by the appropriate dilution of the stock solution using the same solvent. Both stock and working solutions of (*RS*)-9 were stored at 4 °C until used. The stock (1.0 mg/mL ± 0.1) and working solution (50 μg/mL of AC) used as the internal standard were prepared in the same way.

The stability of (*RS*)-9 in rat serum was assessed under a variety of storage and handling conditions using the low-, medium-, and high-quality control (QC) samples. Short-term temperature stability was assessed by analyzing QC samples that had been kept at ambient temperature for 6 h. The stability of samples in autosampler batch was assessed by reanalyzing extracted QC samples kept under the autosampler conditions (10 °C) for 24 h. Long-term stability was assessed over 3 weeks at − 30 °C. Freeze–thaw stability (− 30 °C) was measured over three cycles.

### Pharmacokinetic study

A group of 32 adult male Wistar rats (13–15 weeks old, 200-220 g) were used in the experiment. The animals were purchased from the Animal House at the Faculty of Pharmacy, Jagiellonian University Medical College, Krakow, Poland. During the habituation period, the groups of four rats were kept in a plastic cage at a controlled room temperature (22 ± 2 °C), humidity (55 ± 10%), full-spectrum cold white light (350–400 lx), on 12-h light/12-h dark cycles (the lights came on at 7:00 a.m., and went off at 7:00 p.m.), and had free access to standard laboratory pellets and tap water. (*RS*)-9 dissolved in 1% DMSO in PBS buffer (pH 7.4) were administered intravenously (*iv*) via the tail vein at a dose of 2 mg/kg b.w. Blood samples were collected at 5, 15, 30, 60, 120, 240, and 480 min after compound administration. The blood and tissue samples were collected under general anaesthesia induced by intraperitoneal (*ip*) injection of 50 mg/kg b.w. thiopental. The blood samples were taken into the Eppendorf tubes, allowed to clot and then centrifuged at 3000 ×*g* for 10 min, and serum was collected. The serum samples were immediately frozen at − 30 °C, while the tissue samples (liver, lung, intestine, and kidneys) were stored at − 80 °C until used. All experimental procedures were carried out in accordance with EU Directive 2010/63/EU and approved by the I Local Ethics Committee for Experiments on Animals of the Jagiellonian University in Krakow, Poland (Approval Number 94/2016)*.*

Cumulative urine samples were collected at 0 h (predose) and over the 0–6 h, 6–12 h, and 12–24 h (postdose) course of the study. Faeces samples were collected within 24 h. Urine and faeces samples were stored at − 30 °C until used.

Renal excretion was calculated from cumulative urinary excretion data following compound *iv* administration, and it was expressed as the sum of the amounts excreted over all the collection time intervals, from 0 to 24 h. Renal clearance was calculated using Eq. (1):

1 $$Cl_{r} \; = \;\frac{{A_{e0 - 24} }}{{AUC_{0 - 24} }},$$

where Cl_r_ is the renal clearance; Ae_0-24_ is the total amount of compound excreted in urine within 24 h in unchanged form; AUC_0-24_ is the total area under the compound concentration–time curve measured in serum from 0 to 24 h.

### Preparation of rat liver microsomes

Rat liver microsomes were prepared using a differential centrifugation method. Liver fragments were minced with scissors, washed with TRIS/KCl (pH 7.4) buffer, and homogenised. The homogenate was centrifuged (Sorval WX Ultra Series, Thermo Scientific) at 11500 ×*g* for 20 min at 4 °C. The supernatant (S9 fraction) was transferred to new centrifuge tubes and then ultracentrifuged at 100000 ×*g* for 60 min at 4 °C. The pellet was re-suspended in 0.15 M KCl using Ultra Turrax IKA T10 basic homogeniser (IKA-Werke GmbH & Co. KG Staufen, Germany) and ultra-centrifuged again at 100000 ×*g* for 60 min at 4 °C. The obtained pellet was dispersed in TRIS/sucrose buffer and stored at − 80 °C until used. Protein concentration in microsomal fraction was determined by Lowry protein assay [17].

### In vitro incubation of (*RS*)-9 with rat liver microsomes

The incubation mixture of rat liver microsomes (1.0 mg/mL ± 0.1 of microsomal protein) contained 0.1 M PBS (pH 7.4) and (*RS*)-9 at a concentration of 20 µg/mL dissolved in 1% DMSO in PBS buffer (pH 7.4). The mixture was preincubated for 5 min at 37 °C in a shaking water bath (OLS 200, Grant Instruments, Cambridge, UK). In the next step 10 mM MgCl_2_ and 1 mM NADPH were added to the incubation mixture to initialise the reaction, and samples were further incubated for 90 min. The total volume of the incubation mixture was 500 µL. The incubation of control samples was conducted in the absence of NADPH. Afterwards, the reaction was terminated by the addition of 500 µL of ice-cold acetonitrile containing internal standard (dextrorphan) at a concentration of 250 ng/mL. After centrifugation (3000 ×*g*, 15 min, 4 °C), 100 µL of supernatant was transferred to the inserts, and 25 μL was injected onto the XBridge C18 (30 mm × 2.1 mm i.d. 3 μm, Waters, Ireland) analytical column to determine the concentration of (*RS*)-9 and expected metabolites.

### Sample preparation

A 100 μL volume of serum, urine and homogenates of lung, liver, kidney, intestine, and faeces was pipetted out into 1.5 mL of Eppendorf tubes, and then, 10 μL of the IS working solution (50 μg/mL of AC) was spiked and vortex mixed for 1 min. Next, an aliquot of 1 mL of ethyl acetate was added and then shaken for 20 min (Vibrax, IKA). After vortex mixing, the mixture was centrifuged at 3000 ×*g* for 15 min at 4 °C (Sigma 14-K), and the supernatant (0.5 mL) was first transferred to other tubes and then evaporated to dryness under a stream of nitrogen in a TurboVap LV evaporator. The sec residue was dissolved in 50 μL of a mixture of acetonitrile and water (1:1, v/v), thereafter transferred to the inserts, and 10 μL of the supernatant was injected onto the XBridge C18 (30 mm × 2.1 mm i.d. 3 μm, Waters, Ireland) analytical column.

### LC/ESI–MS/MS analysis

The LC/MS/MS analyses of (*RS*)-9 and expected metabolites using triple quadrupole mass spectrometers API 2000 (Sciex, Concord, Ontario, Canada) coupled to HPLC system Agilent 1100 (Agilent, Waldbronn, Germany) in SRM mode were carried out.

To develop an accurate, valid, and optimal chromatographic condition, the different parameters, including the type of analytical column, the mobile phase composition, and the flow rate of the mobile phase, were examined and compared. Finally, the chromatographic separation of (*RS*)-9 and expected metabolites was conducted using an XBridge C18 analytical column (30 mm × 2.1 mm i.d., 3 μm, Waters, Ireland) thermostated at 30 °C. The flow rate of the mobile phase was set at 0.3 mL/minutes. Eluent A was HPLC grade acetonitrile acidified with 0.1% formic acid, and eluent B was deionised water with the addition of 0.1% formic acid. The elution gradient started with 100% of eluent B, increased for 5 min to 100% of eluent A, maintained 100% of eluent A to 7 min, and then increased to 100% of eluent B for 2 min, and maintained 100% of eluent B to 11 min. Acebutolol was chosen as an internal standard due to its structural similarity to (*RS*)-9 and similar chromatographic behaviour. The sensitivity of the method was poor without formic acid, because a positive ionisation process prefers an acidic condition. Due to a relatively short column and a low flow rate, we could achieve a short separation time with a high column efficiency and a good resolution.

Identification of the precursor ions of the analytes and optimal ionisation conditions using API 2000 mass spectrometer was performed in the full scan mode. Further identification of the most abundant fragment ions and selection of the optimal collision energies and other mass detection parameters were carried out in the product ion scan mode.

The parent and fragment ions and the optimal parameters of an ion source and an ion path for (*RS*)-9 and IS were selected by a flow injection analysis of the analytes at a concentration of 100 ng/mL using the mobile phase of acetonitrile and water (1:1, v/v) with an addition of 0.1% formic acid at a flow rate of 0.2 mL/min. The optimal ion source parameters were as follows: ion spray voltage: 5 kV; source temperature: 500 °C; nebuliser gas: 30 psi; turbo gas: 35 psi; curtain gas: 20 psi; collision gas: 6 psi. Nitrogen (99.9%) from Peak NM20ZA was used as the curtain and collision gas. The parameters of ion path for (*RS*)-9 and IS are seen in Table 1. Because of the similarity in the structure of the parent compound and the metabolites, the same ionisation mechanism and the same parameters of an ion source and an ion path were assumed. For increased sensitivity and selectivity, the MS/MS data acquisition was performed in the selected reaction monitoring (SRM) mode and the mass transitions were *m/z* 357.3 (Q1) → 100.1 (Q3) for (*RS*)-9 and 337.5 (Q1) → 116.2 (Q3) for AC. For expected metabolites, transitions between precursor ions and the most abundant product ions were monitored. The most abundant one used for relative quantification was 373.2 (Q1) → 249.2 (Q3) for M1; 389.4 (Q1) → 325.3 (Q3) for M2; 371.1 (Q1) → 180.7 (Q3) for M3; 533.5 (Q1) → 341.1 (Q3) for M4 and 437.2 (Q1) → 325.3 (Q3) for M5.

**Table 1:** The ion path parameters

Parameters	(*RS*)-9	AC
Declustering potential (V)	50	10
Focusing potential (V)	300	350
Entrance potential (V)	10	10
Collision cell entrance potential (V)	25	10
Collision energy (V)	35	30

### HRMS identification of metabolites

In the first step, the structures of (*RS*)-9 metabolites were previously generated by the software Pallas (Pallas CompuDrug) [18] and MetaSite (Molecular Discovery) [19]. In the next step, the metabolites’ structures were compared with the parent molecule based on the premise that metabolites retain the substructures of the parent compound, undergo a similar MS/MS fragmentation pathway and finally generate product ions and neutral losses associated with those substructures [20]. For this reason, the high-resolution fragmentation mass spectra of metabolites were compared with the specific high-resolution fragments of the parent molecule as a template to interpret the likely pattern of metabolites’ structure. The fragmentation pattern and neutral losses, *e.g*., + 16 Da for hydroxylation (+ O), + 32 Da for dihydroxylation (+ O_2_), + 14 Da for methylation (+ CH_2_), + 42 Da for acetyl conjugation (+ C_2_H_2_O), + 96 Da for sulfation (+ SO_4_), + 176 Da (+ C_6_H_8_O_6_), and + 193 Da (+ C_6_H_9_O_7_) for hydroxyl *O*-glucuronidation have provided evidence of the molecular connectivity of substructures [21].

The metabolites of (*RS*)-9 were identified using a high-resolution mass spectrometer LTQ XL Orbitrap Discovery by sample continuous scanning at a resolution of 30 000, corresponding to a scan time of 200 ms. Orbitrap was calibrated using a mixture of caffeine, MRFA peptide and Ultramark 1621. Conditions of Orbitrap were as follows: spray needle 5 kV; capillary temperature 275 °C; capillary voltage 34.8 V; tube lens voltage 109.7 V; sheath gas (N_2_) 8; auxiliary gas (N_2_) 5. Product ion mass spectra were generated by a collision-induced dissociation of the protonated molecules. Product ion mass spectra were recorded at four different collision energies (10, 20, 30, and 40 eV) using helium (He) as a collision gas. Default automated gain control values for target ions were used for MS and MS/MS analyses. Four decimal monoisotopic masses were used for the mass list and to filter data in Xcalibur software.

### Electrochemical metabolism study

The simulation of the oxidation processes was performed in an electrochemical thin-layer cell equipped with a glassy carbon working electrode (Magic Diamond), a Pd/H_2_ reference electrode, and a counter electrode made of graphite-doped Teflon (ReactorCell^™^, Antec Leyden, Zoeterwoude, The Netherlands). To generate the mass voltammograms, the cell was coupled directly to the QTRAP 5500 mass spectrometer. As a result, a three-dimensional plot called mass voltammogram was obtained, showing the ion intensity as a function of mass-to-charge ratio with the increased potential ramp in the electrochemical cell of the potentiostat. For the electrochemical conversion, the solution of (*RS*)-9 at the concentration of 2 µg/mL in acetonitrile:water (1:1, v/v) with the addition of 0.1% formic acid was passed through the electrochemical cell at a flow rate of 10 µL/min using a syringe pump. Mass spectra were recorded continuously during scanning at the potential from 0 to 2000 mV, and the scan rate of 3.33 mV/s. To obtain more information on (*RS*)-9 oxidation products, additional MS/MS experiments were carried out by applying a constant potential of 1000 mV. Product ion mass spectra of oxidation products were recorded using an enhanced product ion scan mode at different collision energies (10, 20, 30, 40 eV) with a scan range of 50–500 m*/z*.

As (*RS*)-9 is a derivative of carvedilol, the effectiveness of metabolites’ formation by the selected working electrode and the composition of a mobile phase as well as the flow rate were validated using carvedilol as a reference compound.

## Results

### Metabolites’ identification and separation

The stability of the parent compound in stock and working solutions and in vitro in rat serum was investigated before the identification of metabolites. These studies were performed as a part of the method validation for (*RS*)-9. The stock and working solutions were stable during the study in the fridge at 4 °C. Over a 6 h period of the short-term stability test, the predicted concentrations for (*RS*)-9 in serum QC samples deviated within 15% of the nominal concentrations, and no significant degradation was detected in the samples. The data also reflect the stability of the compound during the freezing process. (*RS*)-9 was found to be stable in rat serum when stored at − 30 °C. The results of QC samples following three repeated freeze–thaw cycles have shown that the analyte was stable in the frozen serum at − 30 °C [14].

In the initial step, the identification of (*RS*)-9 metabolites was performed under in silico conditions using Pallas [18] and MetaSite software [19] then in vitro in rat liver microsomes, and in vivo in rat serum, urine, and homogenates of liver, intestines, lungs, kidneys, and faeces. Studies were also performed based on the oxidation and reduction processes of (*RS*)-9 using an electrochemical Roxy^™^ system.

**Fig. 1:**
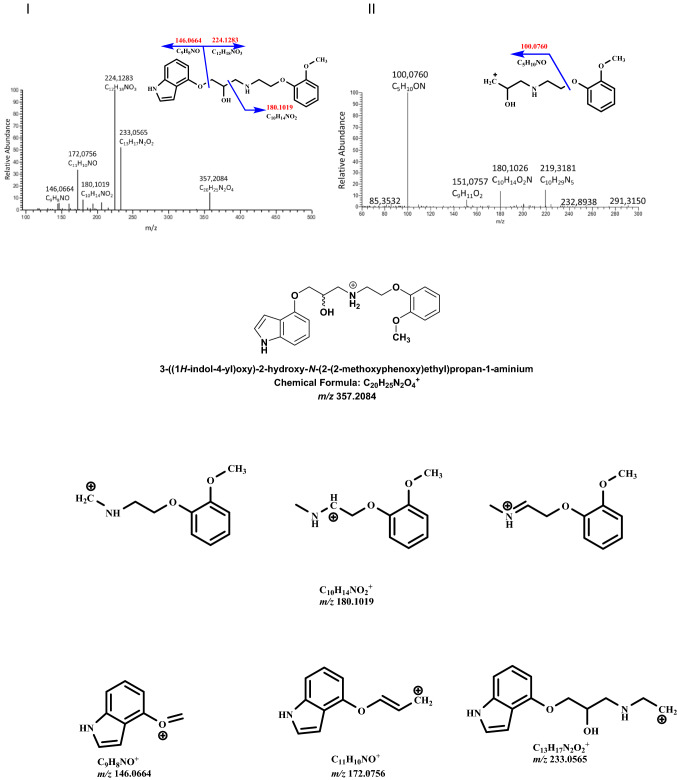
Fragmentation mass spectrum MS2 of (*RS*)-9 (*m/z* 357.2084) (I) and mass spectrum MS3 of the ion *m/z* 224.1283 (II) using He as a collision gas together with likely fragmentation pathways and the structures of the fragments

The product ion mass spectra of protonated (*RS*)-9 (*m/z* 357.2084) recorded at 40 eV collision energy and fragmentation mass spectra MS3 of the ion 224.1283 are shown in Fig. 1 together with proposed structures of the monitored product ions generated by the software SpectraBase [22]. The differences between the theoretically calculated and experimentally determined masses for (*RS*)-9 product ions were very low, less than 2.6 mDa allowed us to define the chemical structure of the metabolites (Table 2).

**Table 2:** Comparison of calculated and found monoisotopic masses for (*RS*)-9 product ions

Elemental composition	Calculated mass (Da)	Found mass (Da)	Deviation (mDa)
C_9_H_8_NO	146.0690	146.0664	−2.6
C_11_H_10_NO	172.0757	172.0756	−0.1
C_10_H_14_NO_2_	180.1020	180.1019	−0.1
C_12_H_18_NO_3_	224.1281	224.1283	+ 0.2
C_13_H_17_N_2_O_2_	233.0540	233.0565	+ 2.5

Metabolites were initially extracted from the total ion chromatogram using *m/z* values. To determine the accurate metabolite signal a mass threshold of 10 ppm was applied. In the next step, the high-resolution fragmentation mass spectra of metabolites were compared with the accurate product ions of (*RS*)-9 [23] using the automated MS/MS comparison tool of ACD/MS Processor [24] and LightSight^™^ Software [25]. The software screens the ion chromatograms of the expected metabolites according to the predicted gains and losses of metabolite molecular masses compared to the molecular mass of the parent compound. For this reason, exact neutral mass loss and product ion mass filters were used to interrogate data (Table 3).

**Table 3:** Nominal molecular masses and product ions for (*RS*)-9 and foreseen metabolites

Compound	*m/z*	Product ions above 5% of relative abundance at 10, 20, 30 and 40 eV collision energy
(*RS*)-9	357	146, 172, 180, 224, 233
M1	373	116, 132, 167, 176, 180, 192, 197, 224, 233, 249, 341
M2	389	164, 180, 219, 325, 341
M3	371	180, 190, 25
M4	533	176, 193, 341, 357
M5	437	160, 172, 180, 341, 357

**Fig. 2:**
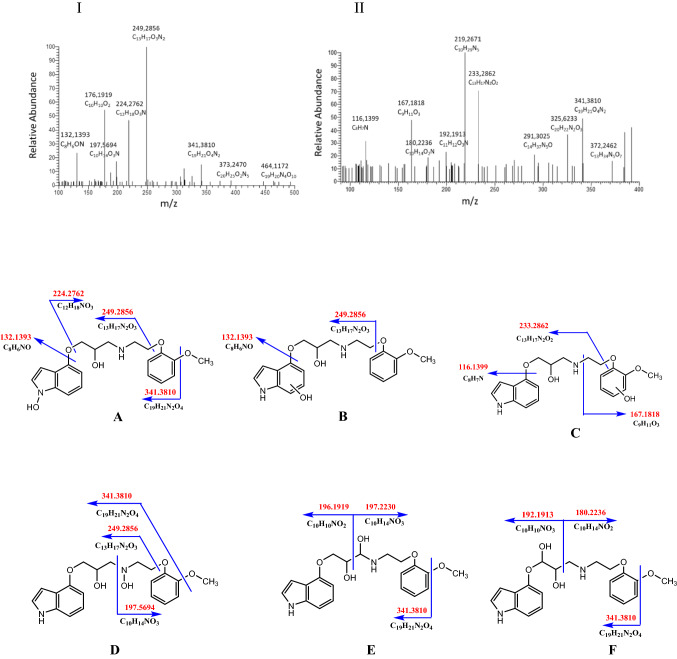
Fragmentation mass spectrum MS2 of metabolite M1 (hydroxylation products, *m/z* 373.2470) (I) and mass spectrum MS3 of the ion *m/z* 341.3810 (II) using He as a collision gas together with likely metabolites (A-F) and fragmentation pathways

Five metabolites of (*RS*)-9 were identified in extracted samples of rat serum, urine, liver, lungs, intestine, kidneys, and faeces. (*RS*)-9 underwent both phase I and phase II metabolism. The processes of *N*-hydroxylation and hydroxylation in position 1 or 3 of the indole group, hydroxylation in position 4 or 5 of the phenoxy group or likely hydroxylation in the aminopropan-2-ol moiety could yield metabolite conventionally known as M1 with *m/z* 373.2470 (Fig. 2).

**Fig. 3:**
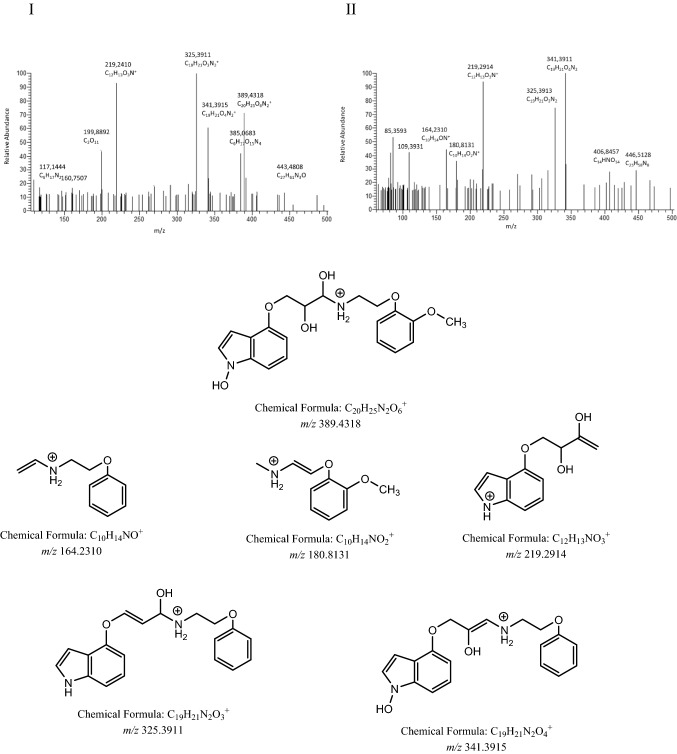
Fragmentation mass spectrum MS2 of metabolite M2 (dihydroxylation products, *m/z* 389.4318) (I) and mass spectrum MS3 of the ion *m/z* 219.2914 (II) using He as a collision gas together with the structures of main fragments

**Scheme 1:**
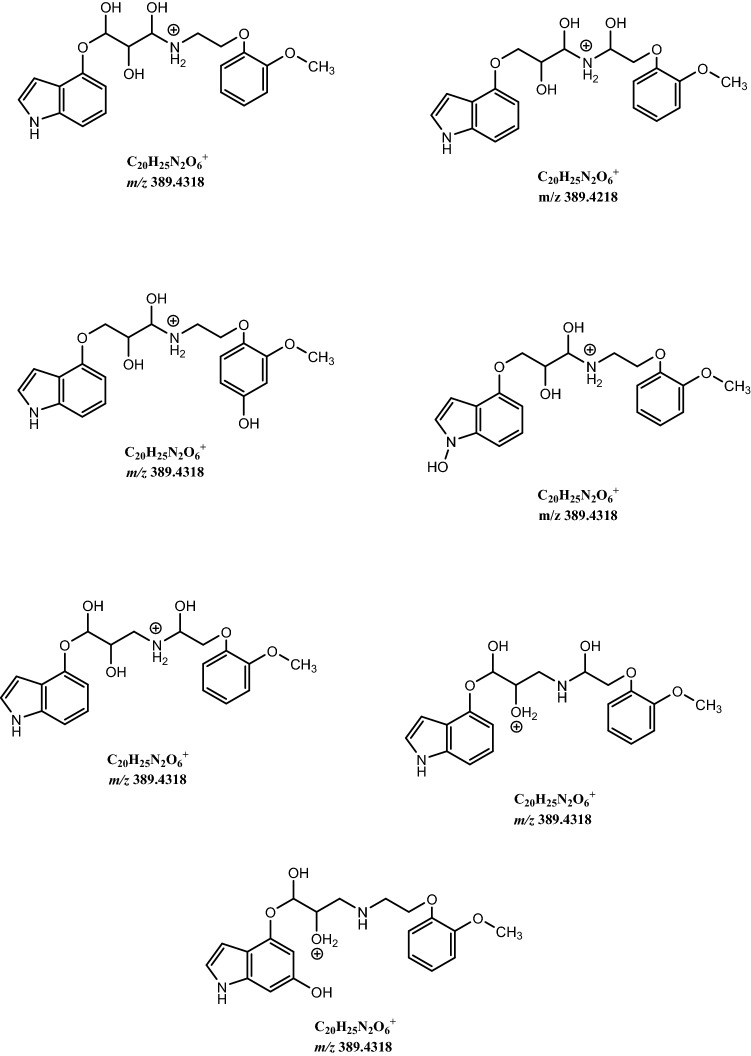
Likely structures of metabolite M2 (*m/z* 389.4318)

As a phase I metabolism, we observed products of dihydroxylation, *e.g.,* one hydroxyl in the indole group and the second in the aminopropan-2-ol moiety conventionally known as M2 (*m/z* 389.4318), Fig. 3 with likely structures seen in Scheme 1.

**Fig. 4:**
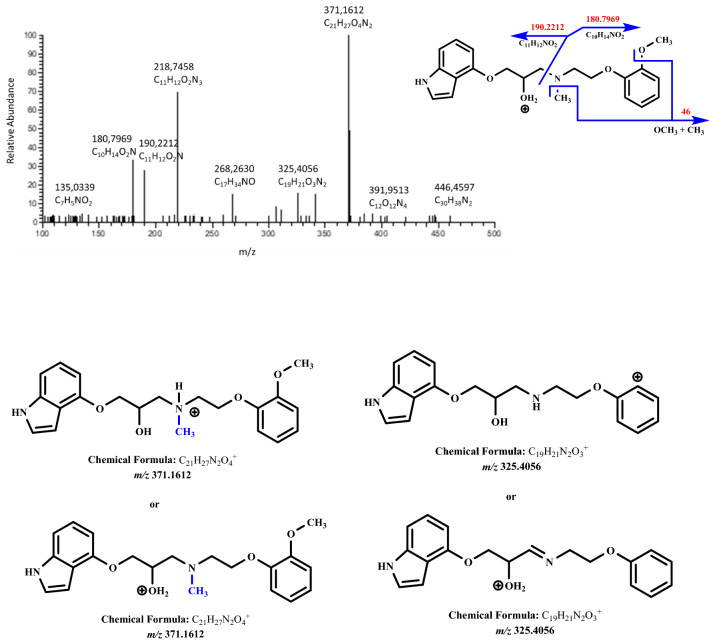
Fragmentation mass spectrum MS2 of metabolite M3 (*N*-methylated compound, *m/z* 371.1612) using He as a collision gas together with likely fragmentation pathways and the structures of the fragments

**Fig. 5:**
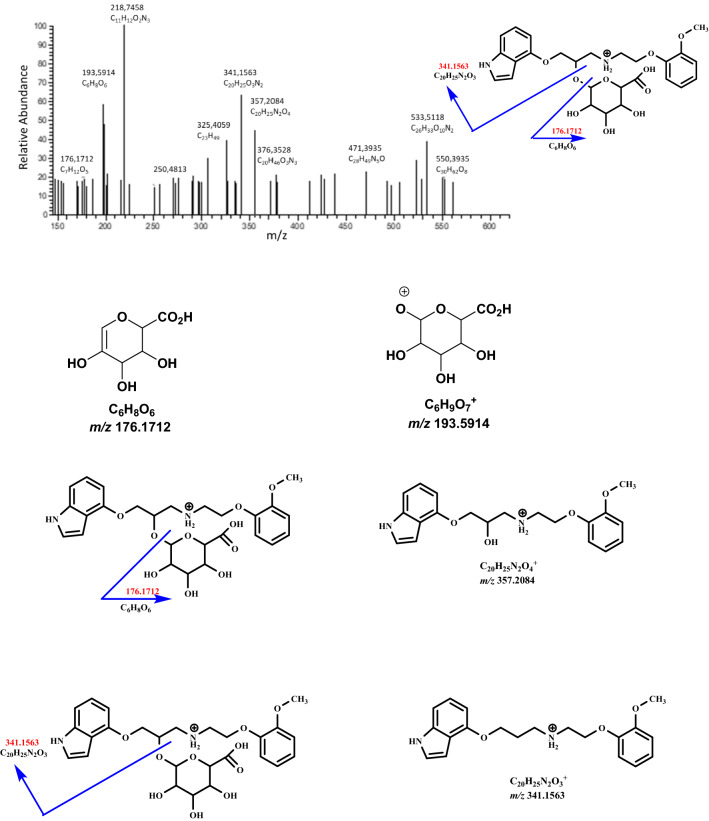
Fragmentation mass spectrum MS2 of metabolite M4 (glucuronide, *m/z* 533.5118) using He as a collision gas together with likely fragmentation pathways and the structures of the fragments

**Fig. 6:**
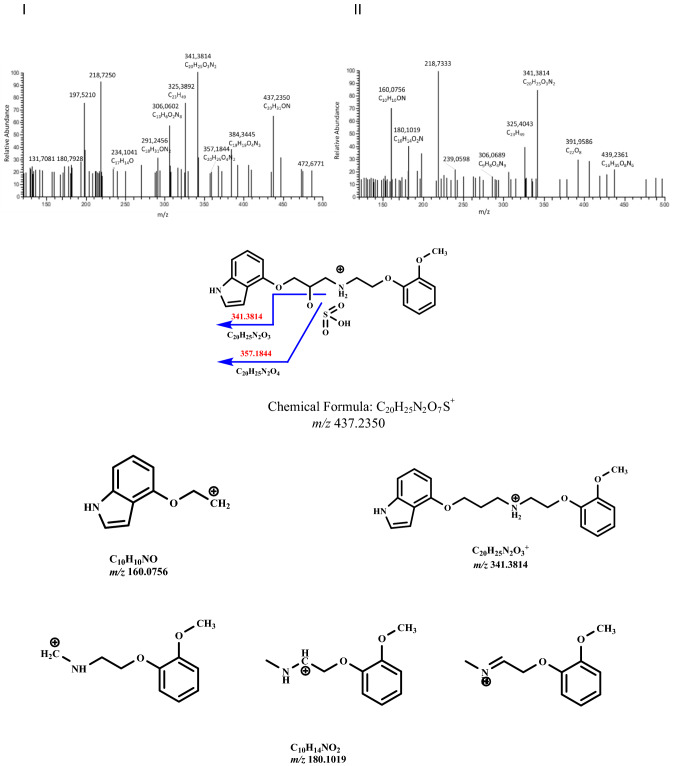
Fragmentation mass spectrum MS2 of metabolite M5 (sulfate, *m/z* 437.2350) (I) and fragmentation mass spectrum MS3 of the ion *m/z* 341.3814 (II) using He as a collision gas together with likely fragmentation pathways and the structures of main fragments

As the major metabolic pathways of phase II biotransformation, we observed the products of *N*-methylation (metabolite M3, *m/z* 371.1612) with fragments *m/z* 180.7994, *m/z* 190.2212, and *m/z* 325.4056 (Fig. 4), *O*-glucuronidation (metabolite M4, *m/z* 533.5118) with characteristic fragments *m/z* 176.1712, *m/z* 193.5914, *m/z* 341.1563, and *m/z* 357.2084 (Fig. 5), and sulfate formation (metabolite M5, *m/z* 437.2350) with fragments *m/z* 160.0756, *m/z* 180.1019, and *m/z* 341.3814 (Fig. 6).

**Fig. 7:**
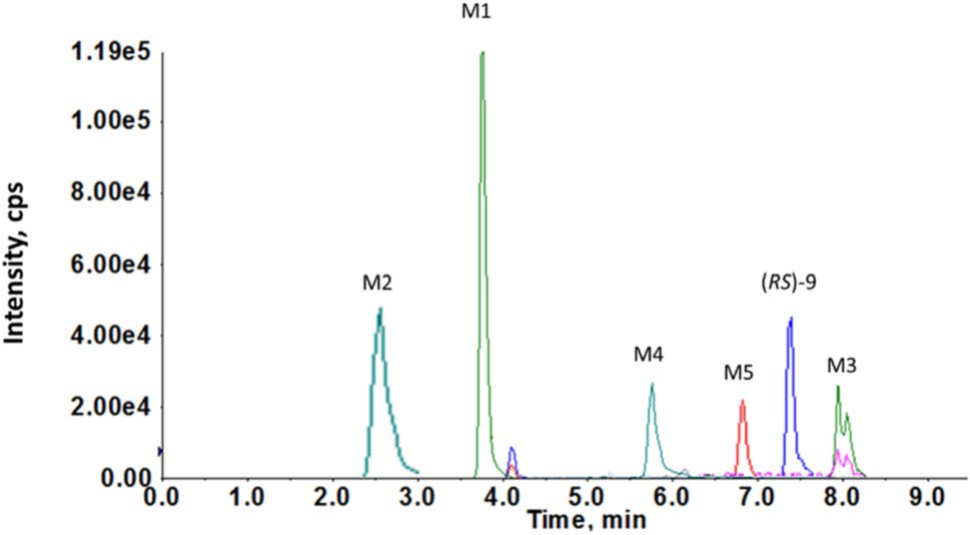
Chromatograms of extracted precursor ions of (*RS*)-9 and likely metabolites recorded in SRM mode in rat serum using LC/MS/MS method

Figure 7 shows the chromatograms of (*RS*)-9 and predicted metabolites registered in SRM mode in rat serum. The elution of (*RS*)-9 and its metabolites was as follows: dihydroxyl derivative (M2, *m/z* 389.4318)–2.7 min, hydroxyl derivative (M1, *m/z* 373.2470)–3.9 min, glucuronide (M4, *m/z* 533.5118)–5.8 min, sulfate (M5, *m/z* 437.2350)–6.8 min. (*RS*)-9 (*m/z* 357.2084) was eluted at 7.4 min, and the most hydrophobic metabolite was the product of methylation (M3, *m/z* 371.1612) eluted at 8 min.

### Electrochemical oxidation

**Fig. 8:**
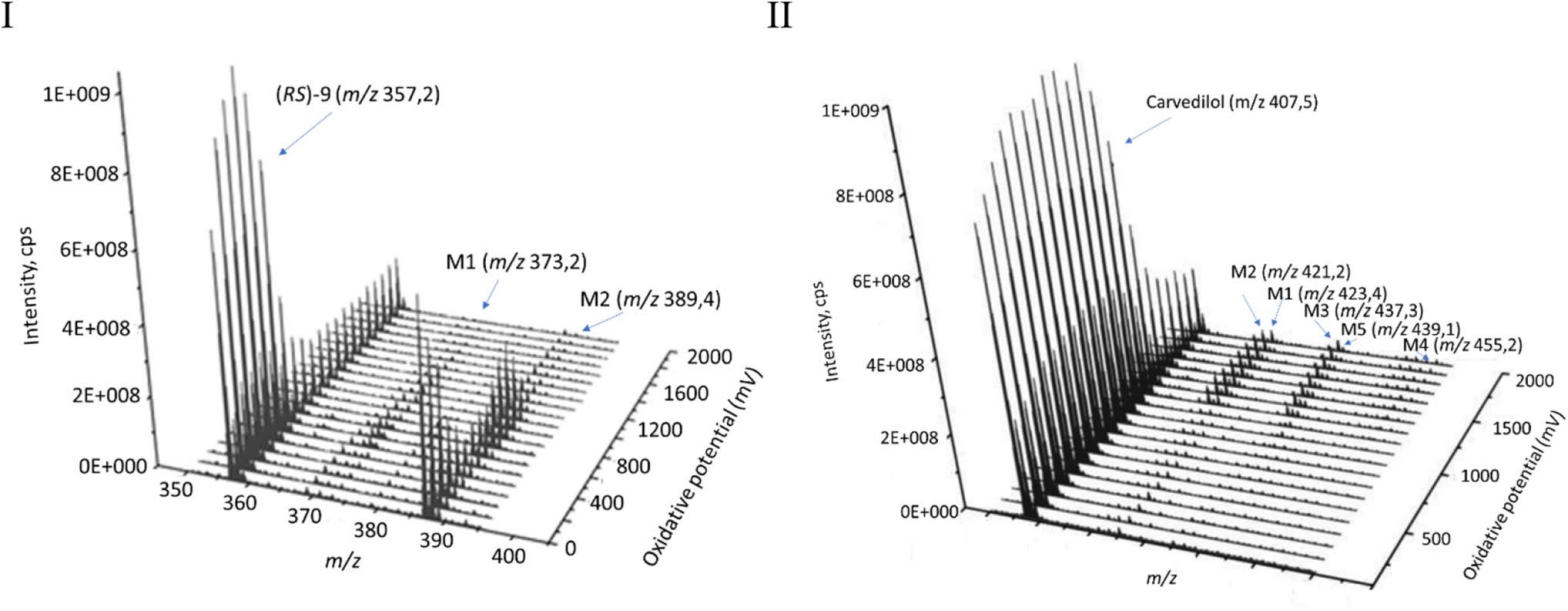
Mass voltammograms for (*RS*)-9 and metabolites M1 and M2 (I) and for carvedilol and metabolites M1–M5 (II) generated by the reaction of oxidation after online EC/ESI–MS analysis showing the varying mass spectra as a function of the oxidative potential

(*RS*)-9 was seen as a protonated ion of *m/z* 357.2. In the mass voltammogram derived from Roxy^™^ system (Fig. 8I), the signal from (*RS*)-9 decreased in a potential of 600 mV with simultaneous increases in the signals of hydroxylation and dihydroxylation products. After fragmentation analysis, two oxidation products were identified: a hydroxylated derivative (M1, + O, *m/z* 373.2, Δ16 Da) and a dihydroxylated product (M2, + 2O, *m/z* 389.4, Δ32 Da). The signal of the dihydroxylation was the most intense, suggesting that this metabolite is the major oxidation product of (*RS*)-9.

The electrochemical Roxy simulation of carvedilol metabolism was used to validate the system and revealed the formation of hydroxy- (*m/z* 423.4), dihydroxy- (*m/z* 439.1), trihydroxy- (*m/z* 455.2), keto- (*m/z* 421.2), and hydroxy-keto (*m/z* 437.3) metabolites (Fig. 8II). The same metabolites of carvedilol identified in vitro using human liver microsomes in the work of Lim H et al. 2007 [26].

### Metabolism of (*RS*)-9 in rat tissues and liver microsomes

Following an *iv* administration of (*RS*)-9, the hydroxyl and dihydroxyl metabolites were the major products, indicating that (*RS*)-9 mainly underwent oxidation and subsequent products were conjugated with glucuronyl, sulfonyl, and methyl groups eliminated in urine and faeces (Table 4). In liver microsomes, only metabolite M1 was identified with M1/(*RS*)-9 peak area ratio 0.32.

**Table 4:** Peak area ratio of metabolites to (*RS*)-9 in rat matrices

Peak area ratio							
Area ratio	Serum	Urine	Faeces	Liver	Intestine	Lungs	Kidneys
M1/(*RS*)-9	1.37	15.9	0.95	2.56	1.45	0.66	0.47
M2/(*RS*)-9	1.23	11.5	0.52	1.72	0.85	0.23	0.20
M3/(*RS*)-9	0.34	0.59	0.62	0.37	0.18	0.26	0.19
M4/(*RS*)-9	1.24	1.05	1.17	0.72	0.65	0.23	0.28
M5/(*RS*)-9	0.24	0.18	0.16	0.14	0.12	0.17	0.21

Renal clearance of (*RS*)-9 was only 0.0014 mL/min, which was only 0.024% of the total clearance of this substance, indicating that the renal route is not the main elimination pathway for this molecule. Whereas the unconjugated excreted metabolites have shown maximum excretion rates between 4 and 12 h after compound intravenous administration, the conjugated metabolites were excreted with maximum rates between 12 and 24 h. Studies of mass balance showed that following a 2 mg/kg b.w. *iv* dose, only approx. 0.1% of (*RS*)-9 was excreted in the urine and faeces within 24 h of post-dose as an unchanged form which may explain the low bioavailability of this compound after intragastric administration [14].

## Discussion

The importance of β-adrenolytic drugs in the treatment of cardiovascular diseases leads to constantly carry out new research for the new compounds with more favourable therapeutic properties. Evaluation of the pharmacokinetic study for (*RS*)-9, an analogue of carvedilol and pindolol, with a particular focus on metabolites, is required for the correct interpretation of its pharmacodynamic effect [12], pharmacokinetic properties [14–16, 27, 28], and ultimately develop a suitable formulation that will provide the best bioavailability of the studied compound. These studies were also dictated by the proven pharmacological activity of metabolites of β-adrenolytics, sometimes in excess of the activity of the parent compound [29, 30].

(*RS*)-9 as a derivative of carvedilol and pindolol may cause similar side effects. Carvedilol, due to its intensive metabolism in the liver, causes a number of adverse effects, including injury of the bile ducts by toxic metabolites, resulting in a mixed-pattern hepatitis with possible cholestatic syndrome and cirrhosis [31]. Due to its extensive hepatic metabolism, carvedilol is contraindicated in severe hepatic impairment [32].

On the contrary, pindolol with its medium lipophilicity and in view of low first-pass effect and moderate metabolism has high bioavailability and low risk of saturation of pharmacokinetic processes and non-linearity [33].

Novel compound (*RS*)-9 was very fast and extensively metabolised into several metabolites in rats. It is believed that aromatic hydroxylation in position 4 or 5 of phenyl ring and position 1 or 3 of the indole ring (*m/z* 373.2470, M1) are involved in phase I metabolism. Furthermore, we identified the products resulting from dihydroxylation (*m/z* 389.4318, M2). Particularly noteworthy are metabolites of phase II biotransformation with *m/z* 371.1612 (methylation product, M3), *m/z* 533.5118 (conjugation with glucuronic acid, M4), and *m/z* 437.2350 (conjugation with sulfuric acid residue, M5).

In the further stage of research, the oxidation behaviour of (*RS*)-9 was studied in a system consisting of an electrochemical cell coupled directly online to a mass spectrometer. As a result, three-dimensional plots called mass voltammograms were obtained, showing the ion intensity as a function of mass-to-charge ratio with the increased potential ramp in the electrochemical cell of potentiostat [34]. The signal from dihydroxylation was the most intense, suggesting that this metabolite is the major oxidation product of (*RS*)-9. The electrochemical method is rapid, simple, sensitive, and inexpensive. The advantage of this method is identification of compounds oxidation products instantly after generation, and therefore, even very reactive and unstable metabolites and intermediates are possible to be generated and detected.

Based on chromatographic peak areas, the hydroxyl and dihydroxyl metabolites were found to be the most intense products, but the glucuronide, sulfate, and methylate of (*RS*)-9 are also of great importance in the metabolic clearance, and they are probably responsible for the incomplete bioavailability of the studied compound after intragastric administration [14]. To estimate the contribution of these metabolites to the biotransformation of (*RS*)-9 and to identify the enzymes responsible for the formation of the metabolites, additional enzyme kinetic studies needs to be done.

Therefore, the knowledge of the metabolites of the compound (*RS*)-9 together with the results of physicochemical, pharmacological, pharmacokinetic, and toxicological properties obtained so far in comparison with the results available for carvedilol and pindolol will allow a better and complete understanding the activity profile of this new molecule.

The identification of metabolites of (*RS*)-9 will accelerate the commercialization of the molecule as a promising new drug with β-blocker activity. The results will also be used for further studies evaluating the potential of ACE2 receptor downregulation, towards the probability of using of β-blockers in the treatment of COVID-19.

## Conclusions

For (*RS*)-9, several major metabolites of phase I (hydroxylated and dihydroxylated products) and phase II (glucuronide, sulfate and methylate forms) were identified. Oxidation processes followed by conjugation reactions seem to be the main biotransformation pathway for (*RS)*-9, possibly contributing to the incomplete bioavailability of this compound as a result of significant presystemic metabolism.

## Abbreviations

Ae_0-24_: Total amount of compound excreted in urine within 24 h in unchanged form
AC: Acebutolol
ACE2 receptor: Receptor for angiotensin-converting enzyme 2
ADME: Absorption, distribution, metabolism, excretion
AUC_0-24_: Total area under the compound concentration–time curve measured in serum from 0 to 24 h
Cl_r_: Renal clearance
FT/MS: Fourier transform mass spectrometry
He: Helium
HPLC: High-performance liquid chromatography system
HRMS: High-resolution mass spectrometry
*ip*: Intraperitoneal injection
*iv*: Intravenous administration
LC/ESI-S/MS: Liquid chromatography–electrospray ionisation tandem mass spectrometry
MgCl_2_: Magnesium chloride
NADPH: Nicotinamide adenine dinucleotide phosphate
NCEs: New chemical entities
(*RS*)-9/2F109: (2*RS*)-1-(1*H*-indol-4-yloxy)-3-((2-(2-methoxyphenoxy)ethyl)amino)propan-2-ol
SARS-CoV-2: Severe acute respiratory syndrome coronavirus 2
SRM: Selected reaction monitoring mode
TRIS: Tris(hydroxymethyl)aminomethane
WHO: World Health Organization
